# Inhibition of *Streptococcus mutans* biofilms with bacterial-derived outer membrane vesicles

**DOI:** 10.1186/s12866-021-02296-x

**Published:** 2021-08-24

**Authors:** Yihui Wang, Joseph P. Hoffmann, Sarah M. Baker, Kerstin Höner zu Bentrup, William C. Wimley, Joseph A. Fuselier, Jacob P. Bitoun, Lisa A. Morici

**Affiliations:** 1 Department of Microbiology and Immunology, Tulane University School of Medicine, 1430 Tulane Ave., SL-38, LA 70112-2699 New Orleans, USA; 2 Department of Biochemistry and Molecular Biology, Tulane University School of Medicine, New Orleans, LA USA; 3 Department of Medicine, Tulane University School of Medicine, New Orleans, LA USA

**Keywords:** Biofilm, Nanoparticle, Bacteria, Burkholderia

## Abstract

**Background:**

Biofilms are microbial communities surrounded by a self-produced extracellular matrix which protects them from environmental stress. Bacteria within biofilms are 10- to 1000-fold more resistant to antibiotics, making it challenging but imperative to develop new therapeutics that can disperse biofilms and eradicate infection. Gram-negative bacteria produce outer membrane vesicles (OMV) that play critical roles in communication, genetic exchange, cargo delivery, and pathogenesis. We have previously shown that OMVs derived from *Burkholderia thailandensis* inhibit the growth of drug-sensitive and drug-resistant bacteria and fungi.

**Results:**

Here, we examine the antibiofilm activity of *Burkholderia thailandensis* OMVs against the oral biofilm-forming pathogen *Streptococcus mutans*. We demonstrate that OMV treatment reduces biofilm biomass, biofilm integrity, and bacterial cell viability. Both heat-labile and heat-stable components, including 4-hydroxy-3-methyl-2-(2-non-enyl)-quinoline and long-chain rhamnolipid, contribute to the antibiofilm activity of OMVs. When OMVs are co-administered with gentamicin, the efficacy of the antibiotic against *S. mutans* biofilms is enhanced.

**Conclusion:**

These studies indicate that bacterial-derived OMVs are highly effective biological nanoparticles that can inhibit and potentially eradicate biofilms.

**Supplementary Information:**

The online version contains supplementary material available at 10.1186/s12866-021-02296-x.

## Background

Biofilms are surface-associated microbial communities surrounded by a complex and highly viscous extracellular polymeric substance (EPS) composed of polysaccharides, proteins, lipids and other microbial-derived products. According to the National Institutes of Health, biofilm-forming pathogens are responsible for 80 % of human infections [1, 2]. Biofilm-related infections often result from microbial colonization of soft tissues or medical implants and can manifest as persistent or chronic diseases [3]. The gel-like EPS encases and protects the microbes from antimicrobials and host immune defense mechanisms, severely obstructing the eradication of biofilm-forming pathogens. Previous work indicates that bacteria within biofilms are 10- to 1000-fold more resistant to common antibiotics [2]. This is due to a number of resistance mechanisms including poor biofilm penetration by antimicrobial agents [4, 5]; metabolically-inactive, dormant, or persister cell bacterial phenotypes with reduced drug susceptibility [6, 7]; and a variety of other bacterial adaptive responses [2, 8, 9]. For these reasons, new therapeutic strategies that can both inhibit and disrupt bacterial biofilms are needed to prevent chronic infections. Various approaches, including inhibitors of EPS formation [10, 11], biofilm-degrading enzymes [12, 13], quorum sensing inhibitors [14], and vaccination [15, 16] are being pursued but none are available for clinical use thus far [17].

Gram-negative bacteria naturally and constitutively shed outer membrane vesicles (OMV) from their surface in an active and selective response to extracellular stressors [18, 19]. OMV nanoparticles range in size between 20 and 450 nm. OMVs contain numerous components including small molecules [20], proteins [21], lipids [22], polysaccharides [23], and RNA/DNA [24, 25] that are embedded within the OMV bi-layered membrane or within the vesicle lumen. OMVs serve various roles in nature, including but not limited to, cargo delivery, cell to cell communication, and genetic exchange [26]. OMVs are ubiquitous and an important particulate constituent of Gram-negative and polymicrobial biofilms [27]. In recent years, several groups, including ours, have reported on the potent antimicrobial activity of OMVs mediated by small molecules, surfactants, and enzymes [28–33]. Given their antimicrobial activity and natural occurrence in bacterial biofilms, we hypothesized that OMVs could potentially be useful in treating or disrupting biofilms formed by competitor bacteria. In previous work, we showed that OMVs derived from *Burkholderia thailandensis* inhibit the growth of drug-sensitive and drug-resistant bacteria and fungi. *B. thailandensis* is an oxidase-positive, Gram-negative rod that is considered largely non-pathogenic to humans. *B. thailandensis* contains a mixture of tetra- and penta-acylated lipid A species that dampens lipopolysaccharide (LPS)-mediated endotoxicity [34]. A number of antimicrobial compounds, including peptidoglycan hydrolases, 4-hydroxy-3-methyl-2-(2-non-enyl)-quinoline (HMNQ), and long-chain rhamnolipid are present in or tightly associate with *B. thailandensis* OMVs [35].

Cariogenic plaque is one of the best-characterized biofilms known to develop within the human body. Although well-described in the literature, it is still a neglected topic and major health problem affecting 60–90 % of children and most adults globally [36]. As one of the most cariogenic microorganisms in dental biofilms, *Streptococcus mutans* is capable of using dietary carbohydrates, especially sucrose, to produce organic acids that demineralize tooth enamel and generate robust biofilms with glucan-based EPS. These biofilms serve as important virulence factors for supporting the bacterial community on dental surfaces [37, 38]. In addition to dental caries, *S. mutans* is also one of the common causes of endocarditis [39]. Furthermore, *S. mutans* is a model Gram-positive organism that can help provide a better understanding of biofilm biology, genetics, and physiology for other closely-related streptococcal as well as other Gram-positive species [40]. In this study, we evaluated the antimicrobial and antibiofilm activity of *B. thailandensis* OMVs against the robust biofilm-forming, oral pathogen *S. mutans*. Here we show that OMVs exhibit potent bactericidal activity against *S. mutans* and reduce total biomass, biofilm integrity, and cell viability when applied to pre-formed *S. mutans* biofilms. Previously identified heat-stable components of OMVs, HMNQ and rhamnolipid, contribute to their antimicrobial and antibiofilm activities. We also observed increased efficacy of the antibiotic gentamicin when it was co-delivered with OMVs. These findings suggest that OMVs may represent a natural resource to combat biofilm-forming microorganisms.

## Results

### Burkholderia thailandensis OMVs exhibit antimicrobial activity against S. mutans

**Fig. 1:**
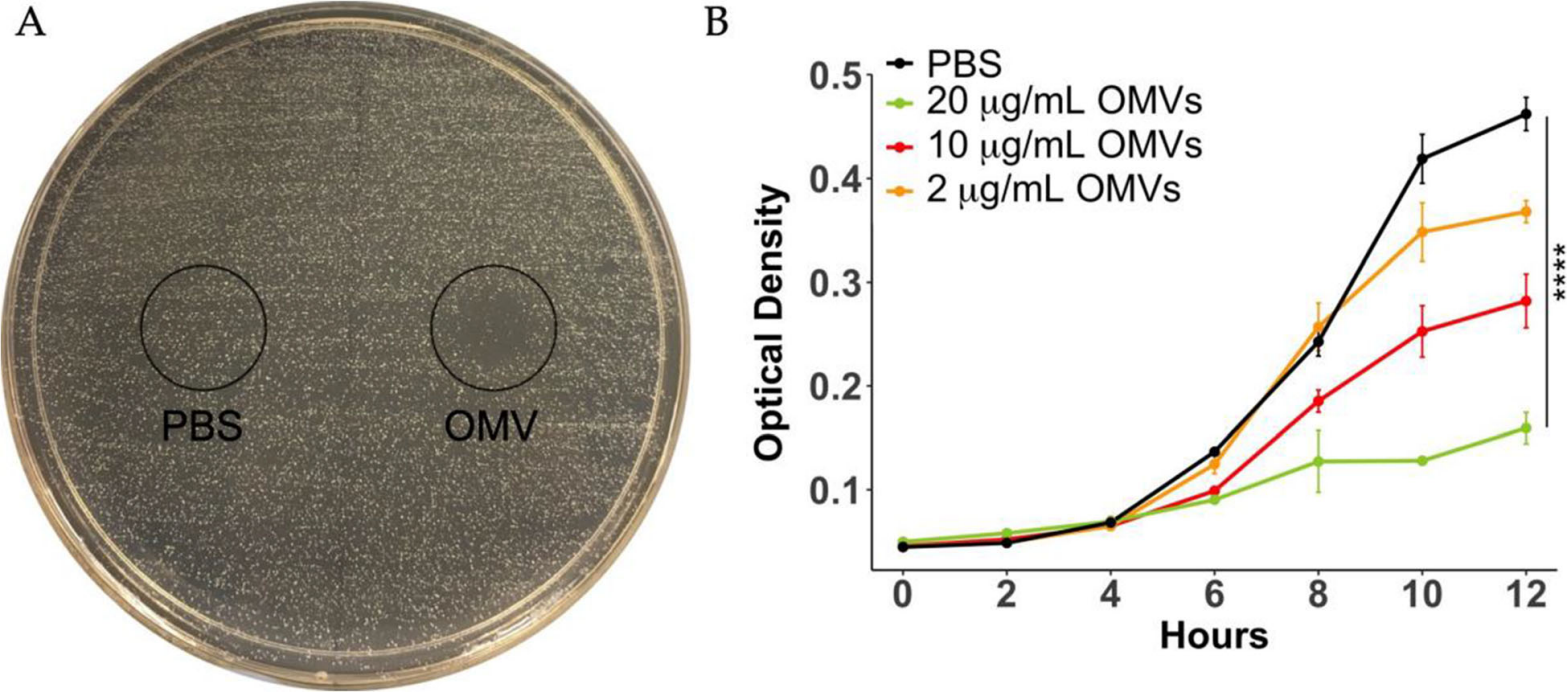
OMVs inhibit the growth of *S. mutans*. The antimicrobial activity of OMVs against *S. mutans* was evaluated on **(A)** agar plates and in **(B)** planktonic cultures. PBS (left side, 10 µL) or OMVs (right side, 10 µg suspended in 10 µL PBS) were spot plated onto agar streaked with *S. mutans* and incubated for 24 h to determine growth inhibition. For planktonic cultures, overnight cultures of *S. mutans* were diluted 1:1000 in broth and treated with 0.2, 1, or 2 µg OMVs or PBS in a total volume of 100 µL. OD_600_ was monitored for up to 12 h. The curves were compared using a Chi-squared test, see Methods. (**** *p* < 0.0001)

OMVs used for the current study were produced from multiple, independent batches and characterized as previously described [35]. We first screened OMV antimicrobial activity against *S. mutans* grown on agar plates and in broth cultures. OMVs inhibited *S. mutans* growth on agar whereas the PBS control treatment did not (Fig. 1 A). We used a Chi-squared test to compare curves at all time points (see Methods). Based on this analysis, growth of *S. mutans* in planktonic cultures was significantly inhibited by OMVs compared to control treatment, and OMV-mediated inhibition was dose-dependent (Fig. 1B).

### OMVs inhibit S. mutans biofilms and planktonic cultures in a time- and dose-dependent manner

**Fig. 2:**
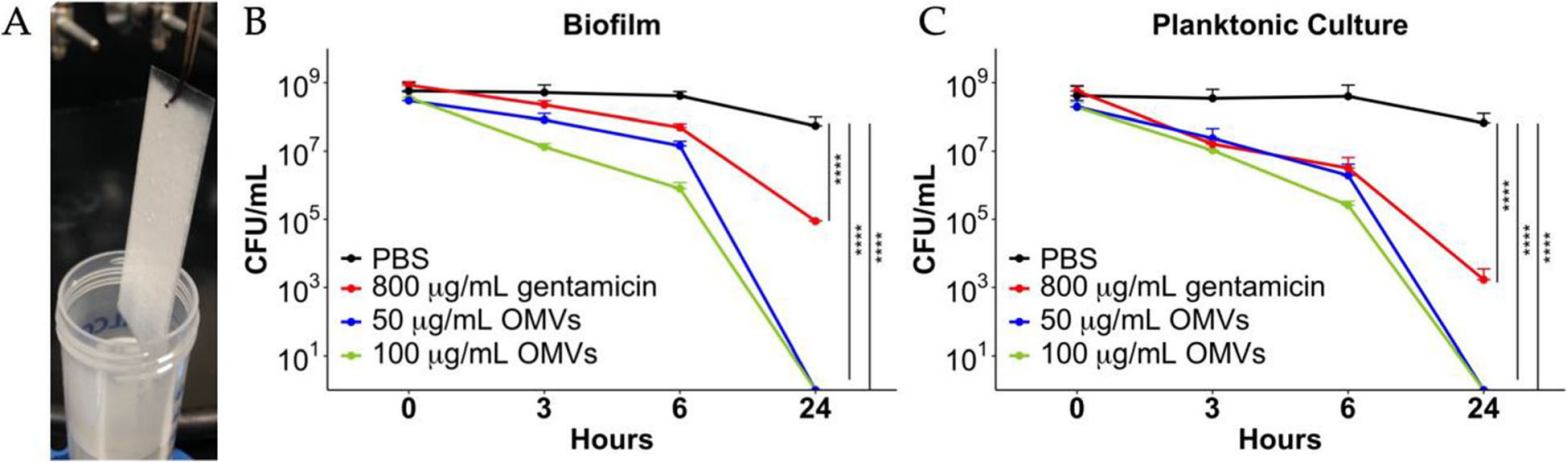
OMVs kill *S. mutans* biofilm and planktonic bacteria in a time- and dose-dependent manner. *S. mutans* biofilm cells were harvested from **(A)** three-day biofilms formed on glass slides cultured in BMGS. Planktonic cells of *S. mutans* were harvested from culture in BHI broth. *S. mutans***(B)** biofilm cells and **(C)** planktonic cells were treated with 50 or 100 µg/mL OMVs, 800 µg/mL gentamicin or PBS for up to 24 h with cell viability monitored. Cells were adjusted to start from the same CFU. Bacterial cells under different treatments were plated on agar for CFU determination at different time points. The curves were compared using a Chi-squared test, see Methods. (**** *p* < 0.0001)

We next evaluated whether OMVs could disrupt biofilm formation by *S. mutans*. *S. mutans* was cultured in biofilm medium containing glucose and sucrose (BMGS) on glass slides for 3 days (Fig. 2 A) then treated them with OMVs, gentamicin, or PBS. All cultures were run in parallel and compared to planktonic overnight cultures receiving the same treatments. The inhibition of *S. mutans* biofilm and planktonic cultures was readily observed within 3 h of OMV treatment with greater than 10-fold reduction in CFU. After 6 h, both biofilm and planktonic cultures treated with OMVs showed 1000-fold reduction in CFU compared to control-treated cultures at the same time-point (Fig. 2B and C). OMV antimicrobial activity was less effective against *S. mutans* biofilms compared to planktonic cultures at 6 h post-treatment. Strikingly, both concentrations tested (50 and 100 µg/ml) OMVs killed all *S. mutans* planktonic and biofilm cultures after 24 h compared to control-treated cultures that still contained more than 10^7^ CFU/mL viable cells. In pilot studies, concentrations less than 50 ug/ml were not effective against biofilms when administered as a single dose. Gentamicin at a concentration of 800 µg/mL failed to clear the bacteria, with more than 10^3^ CFU/mL of viable planktonic cells and 10^5^ CFU/mL of viable biofilm cells remaining after 24 h of treatment (Fig. 2B and C). The Chi-squared test showed highly significant differences between the curves. OMVs displayed potent bactericidal activity against both *S. mutans* planktonic and biofilm cultures in a time- and dose-dependent manner. Notably, while OMVs and gentamicin were both efficient in killing planktonic cells, *S. mutans* biofilms were significantly more susceptible to OMVs than gentamicin.

### OMVs reduce total biomass, biofilm integrity, and cell viability in S. mutans biofilms

**Fig. 3:**
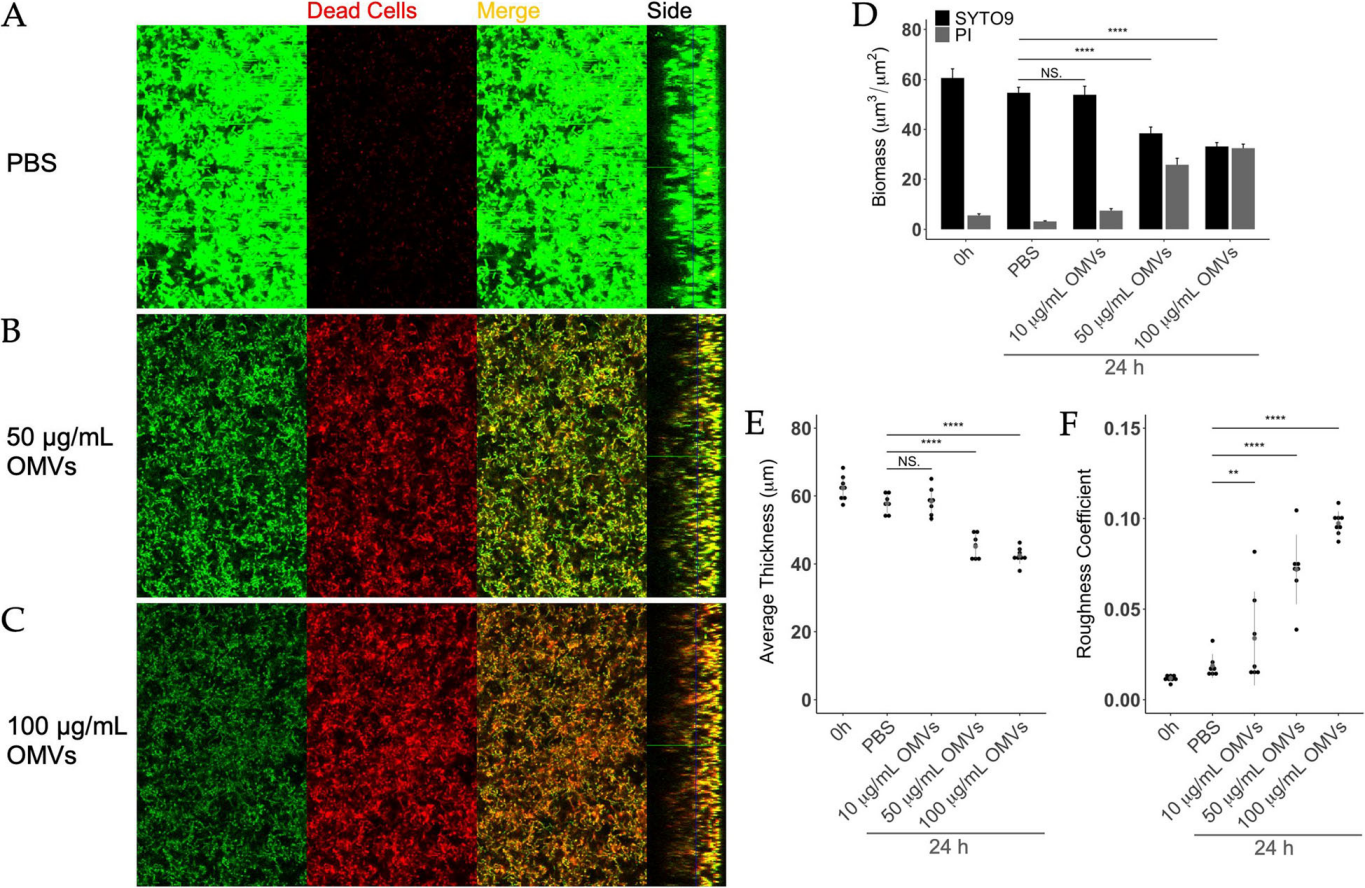
OMVs exhibit bactericidal and antibiofilm activities on *S. mutans*. *S. mutans* biofilms were grown on chamber-slides in BMGS for three days before treatment with **(A)** PBS, **(B)** 50 µg/mL, or **(C)** 100 µg/mL OMVs for 24 h. Following staining with LIVE/DEAD *Bac*Light fluorescent dye (SYTO 9/propidium iodide), biofilms were subjected to optical dissection using confocal microscopy. Green: total biomass. Red: dead cells. Side bars indicate the thickness of the biofilms. Post-acquisition analyses of the fluorescent images were performed using COMSTAT 2.0. Treatments with OMVs or PBS were compared for **(D)** total biofilm biomass/dead cell biomass, **(E)** average thickness, and **(F)** roughness coefficient. Roughness coefficients of the biofilms were calculated as an indication of biofilm integrity. The results were analyzed using one-factor ANOVA. (** *p* < 0.01, **** *p* < 0.0001, NS = not significant)

To further evaluate OMV-mediated disruption of pre-formed, intact *S. mutans* biofilms, we utilized fluorescent confocal microscopy combined with COMSTAT analysis. Biofilms of *S. mutans* were grown on chamber slides for 3 days then treated with PBS or increasing doses of OMVs for 24 h prior to imaging. Biofilms were stained with SYTO 9 to examine total biomass and propidium iodide (PI) to determine dead cell biomass. Remarkably, increasing concentrations of OMVs led to a reduction in total biomass (green fluorescence) in *S. mutans* biofilms as well as an increase in the biomass of dead cells (red fluorescence; Fig. 3 A-D). There was also an observable decrease in biofilm thickness after OMV treatments (Side bars, Fig. 3 A-C). Furthermore, the lack of red fluorescence at the bottom of OMV-treated biofilms suggests an inability to fully penetrate the biofilm, which is one of the obstacles in developing effective antibiofilm agents. COMSTAT analysis indicated that OMVs reduced biofilm thickness and integrity in a dose-dependent manner (Fig. 3E). The significant increase in roughness coefficient is further evidence of an overall deterioration of biofilm integrity (Fig. 3 F) [35, 41]. Scanning electron micrographs of OMV-treated biofilms corroborated the COMSTAT analyses by revealing differences in biofilm architecture. In particular, OMV-treated biofilms had a diminished presence of extracellular material and structures compared to healthy control biofilms (Supplemental Figure 1).

### Heat-inactivated OMVs exhibit antimicrobial and antibiofilm activity against S. mutans

**Fig. 4:**
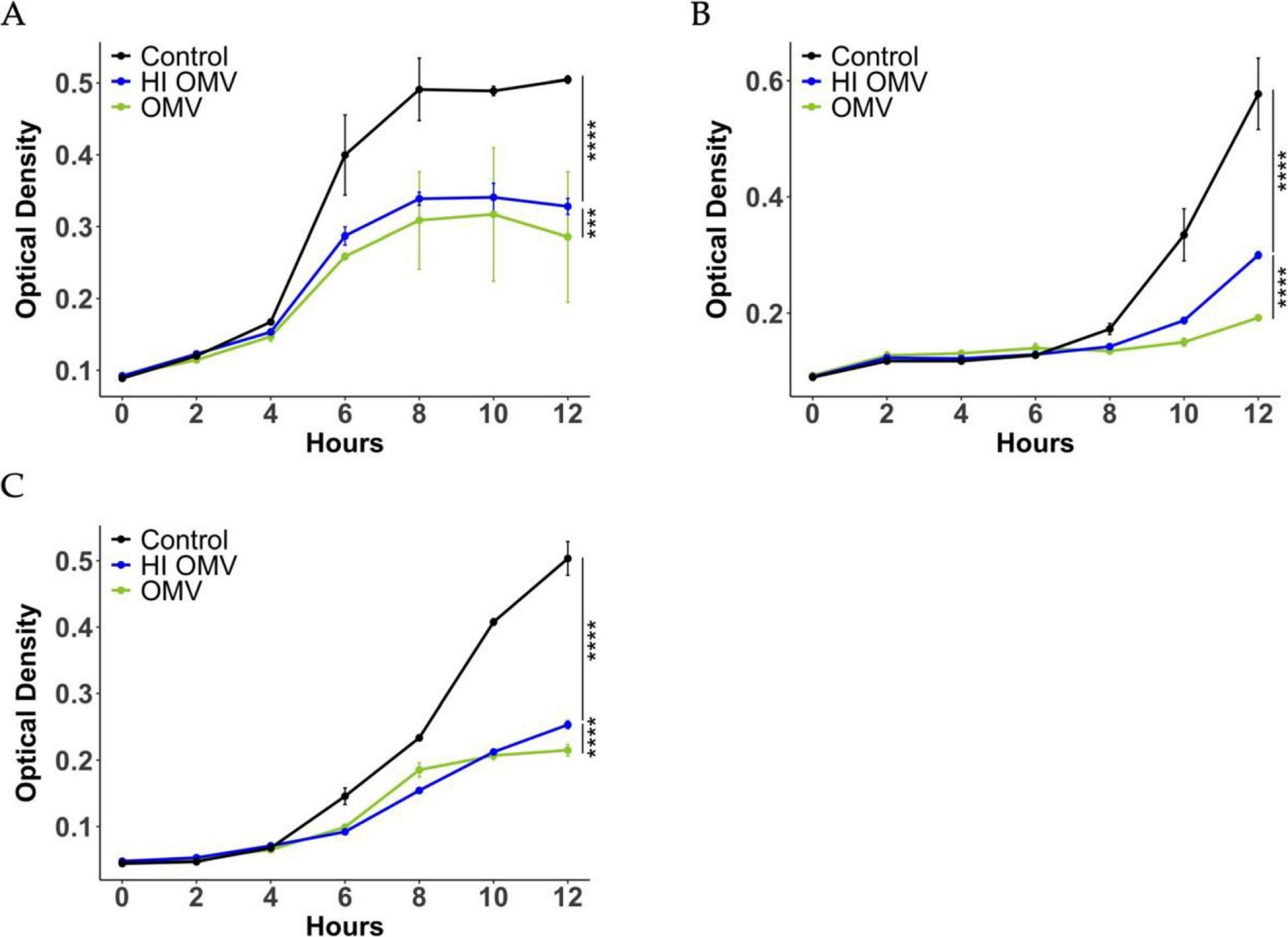
Heat-inactivated OMVs display antimicrobial activity. Heat-inactivated OMVs inhibit the growth of *S. mutans***(A)** UA159 strain **(B)** Clarke strain and **(C)** OMZ175 strain. Overnight cultures of *S. mutans* were diluted 1:100 in broth and treated with PBS, 2 µg OMVs, or 2 µg heat-inactivated OMVs in a total volume of 100 µL. OD_600_ was monitored for up to 12 h. The curves were compared using a Chi-squared test, see Methods. (*** *p* < 0.001, **** *p* < 0.0001)

**Fig. 5:**
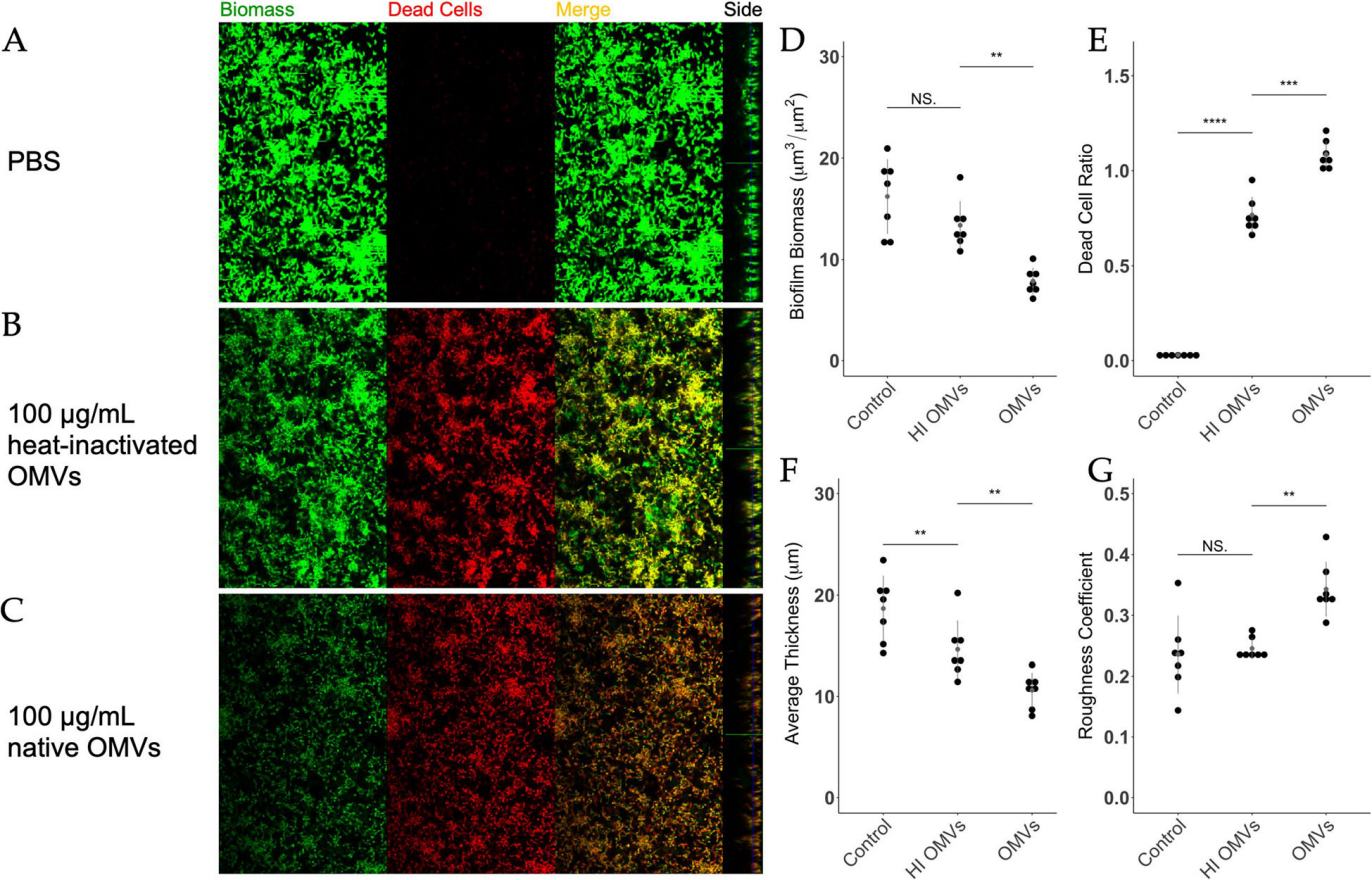
Heat-inactivated OMVs exhibit bactericidal and antibiofilm activities against *S. mutans. S. mutans* biofilms were grown on chamber-slides in BMGS for 24 h before being treated with **(A)** PBS, **(B)** 100 µg/mL heat-inactivated OMVs, or **(C)** 100 µg/mL native OMVs for another 24 h. After staining with LIVE/DEAD *Bac*Light fluorescent dye (SYTO 9/propidium iodide), biofilms were subjected to optical dissection using confocal microscopy. Green: total biomass. Red: dead cells. Side bars indicate the thickness of the biofilms. Post-acquisition analyses of the fluorescent images were performed using COMSTAT 2.0. Treatments with heat-inactivated OMVs, native OMVs, or PBS were compared for **(D)** biofilm biomass, **(E)** dead cell ratio, **(F)** average thickness, and **(G)** roughness coefficient. Roughness coefficients of the biofilms were calculated as an indication of biofilm integrity. The results were analyzed using one-factor ANOVA. (* *p* < 0.05, ** *p* < 0.01, *** *p* < 0.001, **** *p* < 0.0001, NS = not significant)

In previous work, we demonstrated that *B. thailandensis* OMVs contain heat-labile peptidoglycan hydrolases that contribute to their antimicrobial activity against *Staphylococcus aureus* by degrading the cell wall [35]. However, when OMVs were applied to purified *S. mutans* peptidoglycan, OMVs failed to degrade the peptidoglycan (not shown). This suggested that OMV antimicrobial activity against *S. mutans* does not depend on peptidoglycan degrading enzymes. To further examine this, OMVs were heat-inactivated to destroy enzymatic activity. When applied to *S. mutans* planktonic cultures, heat-inactivated OMVs still significantly inhibited the growth of three different *S. mutans* strains, including low passage isolates, albeit to a lesser extent than native OMVs (Fig. 4). To investigate the antibiofilm activity of heat-inactivated OMVs, *S. mutans* biofilms were pre-formed on chamber slides then treated with PBS, 100 µg/mL heat-inactivated OMVs, or 100 µg/mL native OMVs. Compared to PBS-treated control, treatment with heat-inactivated OMVs significantly decreased the average thickness and increased the dead cell ratio of pre-formed biofilms but did not affect the total biomass or roughness coefficient (Fig. 5). Thus, both heat-labile and heat-stable components of OMVs appear to contribute to anti-biofilm activity (Fig. 5).

### Rhamnolipid and HMNQ exhibit bactericidal and antibiofilm activity against S. mutans

**Fig. 6:**
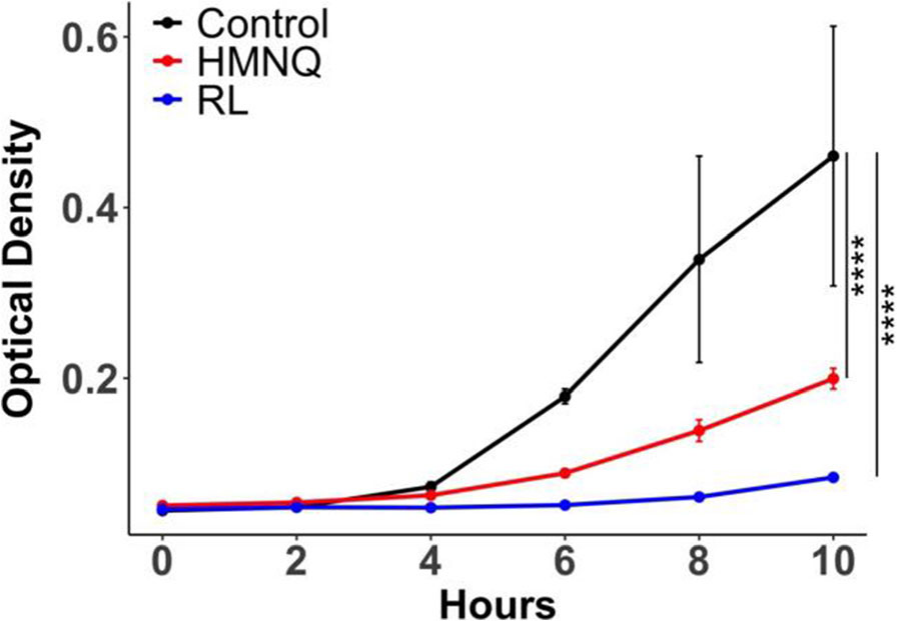
HMNQ and rhamnolipid inhibit the growth of *S. mutans*. Overnight cultures of *S. mutans* were diluted 1:1000 in broth and treated with PBS, 100 µM HMNQ, or 64 µg/mL rhamnolipid (purified from *B. thailandensis* OMVs) in a total volume of 100 µL. OD_600_ was monitored for 12 h. The curves were compared using a Chi-squared test, see Methods. (**** *p* < 0.0001)

**Fig. 7:**
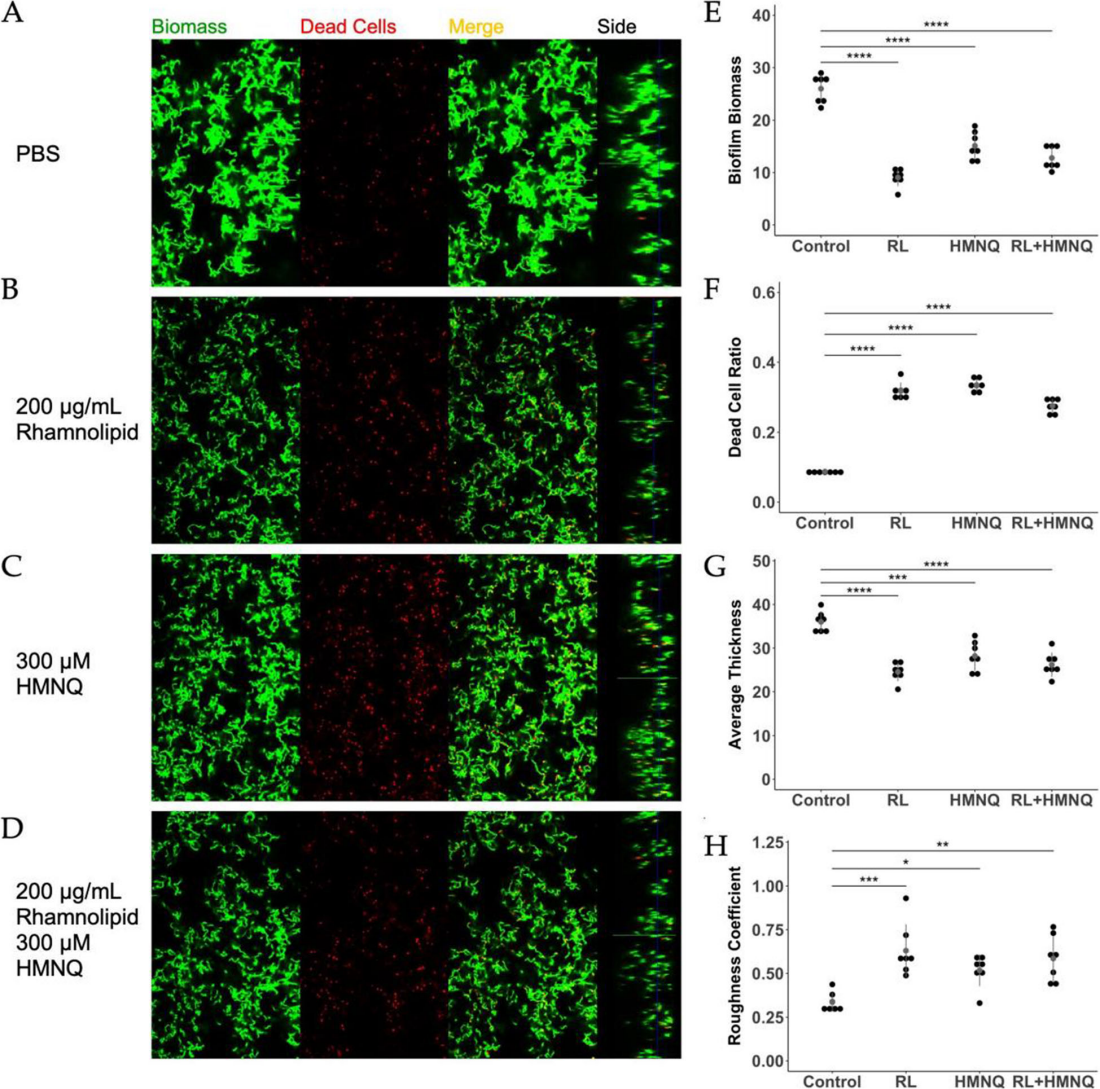
HMNQ and rhamnolipid exhibit bactericidal and antibiofilm activities against *S. mutans*. *S. mutans* biofilms were grown on chamber-slides in BMGS for 24 h before being treated with **(A)** PBS, **(B)** 200 µg/mL rhamnolipid, **(C)** 300 µM HMNQ, or **(D)** a combination of 200 µg/mL rhamnolipid with 300 µM HMNQ for another 1.5 h. After Staining with LIVE/DEAD *Bac*Light fluorescent dye (SYTO 9/propidium iodide), biofilms were subjected to optical dissection using confocal microscopy. Green: total biomass. Red: dead cells. Side bars indicate the thickness of the biofilms. Post-acquisition analyses of the fluorescent images were performed using COMSTAT 2.0. Treatments with heat-inactivated OMVs, native OMVs, or PBS were compared for **(E)** biofilm biomass, **(F)** dead cell ratio, **(G)** average thickness, and **(H)** roughness coefficient. Roughness coefficients of the biofilms were calculated as an indication of biofilm integrity. The results were analyzed using one-factor ANOVA. (* *p* < 0.05, ** *p* < 0.01, *** *p* < 0.001, **** *p* < 0.0001)

Previously, we identified the heat-stable components, rhamnolipid and HMNQ, that contributed to *B. thailandensis* OMV-mediated antimicrobial activity against various bacteria and fungi, including drug-resistant pathogens [35]. Next, we evaluated the antimicrobial activity of purified rhamnolipid and HMNQ on planktonic *S. mutans*. The purified compounds were added to broth cultures of *S. mutans* as was done for OMVs using concentrations previously shown to be effective against other bacteria [35]. Rhamnolipid and HMNQ significantly inhibited the growth of the bacteria (Fig. 6). When applied to pre-formed *S. mutans* biofilms, rhamnolipid (Fig. 7B) and HMNQ (Fig. 7 C) significantly decreased the total biomass and biofilm thickness (Fig. 7E,G) and increased the ratio of dead cells in the biofilms as well as the roughness coefficient (Fig. 7 F,H), as compared to PBS control (Fig. 7 A, E-H). No additive effect was observed when *S. mutans* biofilms were treated with a mixture of HMNQ and rhamnolipid (Fig. 7D-H).

### OMVs enhance antibiotic efficacy against S. mutans biofilms

Several strategies have been proposed to target drug-resistant biofilm-related infections including combination therapies. To determine if OMVs could enhance antibiotic efficacy, we examined the effect of OMVs with gentamicin, which is commonly used in combination with other antibiotics against *S. mutans* infections [42]. We first established that the minimal inhibitory concentration (MIC) for gentamicin against *S. mutans* biofilms (MIC-B) was 7-fold higher than the MIC for planktonic cultures (MIC-P) (Table 1). When the antibiotic treatment was supplemented with sub-inhibitory concentrations of OMVs at 5, 10, and 20 µg/mL, the MIC-B of gentamicin decreased by 2-, 4-, and 14-fold (Table 1), respectively, indicating an enhanced treatment effect upon co-administration with OMVs.

**Table 1:** *B. thailandensis* OMVs enhance efficacy of gentamicin against *S. mutans* biofilms

Gentamicin MIC (µg/mL)	OMVs (µg/mL)			
	**0**	**5**	**10**	**20**
Biofilm	141.42	62.98	35.36	9.92
Planktonic	20.00	-	-	-

^1^ Planktonic cultures and biofilms of *S. mutans* were treated with 2-fold serial dilutions of gentamicin alone or gentamicin combined with 5, 10, 20 µg/mL OMVs for 24 h. Fresh BHI was added and incubated for another 24 h to allow the detachment of viable cells within biofilms. OD_600_ was measured before and after the incubation to show the inhibition of bacterial growth. MICs were calculated as the geometric mean from the replicates

## Discussion

In this study, we investigated the antimicrobial activity of *B. thailandensis* OMVs against a well-characterized biofilm-forming pathogen *S. mutans*. *S. mutans* forms highly robust biofilms when cultured in BMGS, allowing us to evaluate the antibiofilm properties of OMVs in this well-established model system. We show that OMVs are bactericidal against both planktonic and pre-formed *S. mutans* biofilms. OMV treatment reduced the overall biomass, biofilm integrity, and cell viability and altered the cellular morphology of *S. mutans* biofilm cells in a dose-dependent manner. Heat-inactivation of OMVs partially reduced the effect, suggesting that heat-labile components contribute to the disruption of *S. mutans* biofilms. It appears that peptidoglycan hydrolases present in the OMVs do not contribute to the antimicrobial activity against *S. mutans* since OMVs failed to degrade purified *S. mutans* peptidoglycan. This is in contrast to our previous observations that demonstrated OMVs degraded peptidoglycan derived from *S. aureus* [35]. Peptidoglycan is the major component of the bacterial cell wall and is quite diverse in terms of sugar and peptide composition and length among bacterial species [43], which could potentially explain the inability of peptidoglycan hydrolases from *B. thailandensis* OMVs to recognize *S. mutans*. Proteomic analysis identified more than one hundred proteins present in *B. thailandensis* OMVs (data not shown), and it is plausible that one or more of these contribute to OMV antimicrobial activity, warranting further study.

We previously identified HMNQ and rhamnolipid as broad-spectrum antimicrobial compounds that were present in *B. thailandensis* OMVs [35]. Here, were show that both of these compounds exhibit antimicrobial and antibiofilm activity against *S. mutans*. HMNQ is the dominant form of 4-hydroxy-2-alkylquinolines (HAQs) family molecules produced by *B. thailandensis* [44]. It has been shown to function as an ionophore, to disrupt proton motive force, and to inhibit pyrimidine biosynthesis in *Escherichia coli* [45]. Rhamnolipids are well studied in *P. aeruginosa* [46] and the antimicrobial and antibiofilm activities of rhamnolipids have been previously characterized [47–49]. Interestingly, rhamnose is used for the synthesis of cell wall antigen rhamnose-glucose polysaccharide (RGP) and genetic ablation of either rhamnose biosynthesis gene *rmlD* and rhamnose-glucose polysaccharide gene *rgpG* in *S. mutans* severely affects biofilm formation and cell wall turgor [50]. While anabolic pathways for rhamnose biosynthesis have been described, catabolic pathways for rhamnose degradation have yet to be described in *S. mutans*. It’s possible that OMVs present high concentrations of unusable rhamnose in the form of rhamnolipids which alters RGP synthesis and subsequent biofilm formation. Current studies are underway to investigate the impact of OMVs on rhamnose availability and utilization in *S. mutans*. Interestingly, HMNQ and rhamnolipid alone or in combination were less effective than intact OMVs in disrupting *S. mutans* biofilms. This may be a consequence of concentration or could indicate that OMVs are better able to penetrate the biofilm. In support of the latter explanation, the efficacy of gentamicin against *S. mutans* biofilms was enhanced when the antibiotic was co-delivered with OMVs. This corroborates previous work that demonstrated a synergistic bactericidal effect of gentamicin-containing *P. aeruginosa* OMVs [30, 32]. Together, our results indicate that OMVs derived from certain bacteria may be a viable adjunct therapy to enhance the effectiveness of antibiotics against bacterial biofilms. They could also be used in conjunction with other new antibiofilm therapeutics, such as recently described antimicrobial peptides D-CONGA and GH-12 that display antibiofilm activities [51, 52].

*S. mutans* forms one of the most robust biofilms in nature. The ability of bacterial-derived OMVs to inhibit and disrupt pre-formed *S. mutans* biofilms and to enhance antibiotic efficacy are compelling findings. These results suggest that vesicle-based strategies could lead to effective antibiofilm treatments. Compared to other nanoparticles used in clinical or industrial applications, naturally-derived OMVs may provide certain advantages in terms of lipid composition and cargo packaging. Studies characterizing phospholipids in *P. aeruginosa* OMVs showed a higher membrane rigidity of the vesicles compared to their bacterial cell outer membrane, which makes them more resistant to environmental perturbation [22]. OMVs are relatively stable at room temperature and resistant to freeze-thaw cycles, which makes them suitable for lyophilization and confers a broad array of applications [53]. Furthermore, bacterial secretory cargo is protected from degradation in the environment by selective packaging into OMVs [53]. Through selective culturing methods or genetic engineering, OMVs could be enriched with specific cargo that could work synergistically against different microbial species and complex biofilms. For clinical applications, careful consideration must be given to the presence of endotoxin in OMVs derived from bacterial species with highly inflammatory lipid A moieties [54]. Our work suggests that the soil bacterium *B. thailandensis* which possesses a weakly stimulatory lipid A secretes multiple antimicrobial agents through OMVs that target diverse microbes. Future studies could evaluate OMV genetic modifications to lower their toxicity while improving their antimicrobial potency and specificity.

## Conclusions

Bacterial biofilms associated with human diseases, such as dental plaque caused by *S. mutans*, can be very difficult to eradicate. Here we used well-established *S. mutans* biofilm models combined with imaging and COMSTAT computer program analysis to study the antibiofilm activity of bacterial-derived outer membrane vesicles. We demonstrate the remarkable ability of OMVs to disrupt *S. mutans* biofilms and to enhance the efficacy of a co-delivered antibiotic. OMVs represent a promising microbial-derived product for the treatment or inhibition of biofilm-forming species.

## Methods

### Bacterial Strains and Growth Conditions

*B. thailandensis* strain E264 and *S. mutans* strains Clarke and UA159 (low passage isolate) were obtained from ATCC. *S. mutans* OMZ175 is a low passage clinical isolate that can invade epithelial cells (kindly provided by Dr. Tom Wen, LSU School of Dentistry). *B. thailandensis* was propagated in lysogeny broth (LB) and incubated at 37 °C with 233 rpm oscillation. *S. mutans* was cultured in brain heart infusion broth (BHI), while solid medium was made by adding 1.5 % (wt/vol) agar. *S. mutans* cultures were maintained at 37 °C in an aerobic chamber containing 5 % CO_2_ under static conditions.

### OMV Purification

For OMV preparation, *B. thailandensis* E264 was grown in 3 L LB broth at 37 °C until late-log-phase (18 h). The intact bacteria were pelleted by centrifugation (Thermo Scientific, Sorvall RC5C plus) at 6,000⋅ *g* for 60 min at 4 °C, and the supernatant was removed and filtered through a 0.22 μm polyethersulfone (PES) filter (Millipore) to remove any remaining bacteria or large bacterial fragments. To ensure the supernatant was free of viable bacteria, one milliliter of filtered supernatant was streaked onto *Pseudomonas* isolation agar and incubated for 48 h at 37 °C. The OMVs were precipitated by slowly adding solid ammonium sulfate while stirring gently until a molarity of 1.5 was reached. OMVs were allowed to precipitate overnight at 4 °C before being harvested by centrifugation at 11,000⋅ *g* for 45 min at 4 °C. The resulting pellet, consisting of crude vesicles, was resuspended with 8 mL 60 % sucrose in 30 mM Tris-HCl pH 8.0 (wt/vol), which was filter sterilized through a 0.22 μm PES filter and layered at the bottom of a centrifuge tube. A sucrose gradient was prepared in centrifuge bottles by slowly layering 5 mL 55 %, 5 mL 50 %, 4 mL 45 %, 4 mL 40 %, and 4 mL 35 % sucrose in 30 mM Tris-HCl pH 8.0 over 4 mL of the crude OMV preparation. The sucrose gradient with crude OMVs was ultracentrifuged (Beckman Coulter, Optima XL-100 K) at 200,000⋅ *g* for 3 h at 4 °C. Equal 3 mL fractions were removed sequentially from the top and stored at 4 °C. To ensure the purity of the fraction, 200 µL of each was precipitated with 20 % Tri-chloroacetic acid (TCA) and washed twice with acetone. The resulting protein pellets were run on an SDS-PAGE gel (4–20 %, Bio-Rad). The final OMV preparation was recovered by pooling the purest fractions in 30 mM Tris-HCl pH 8.0 followed by ultracentrifugation at 200,000⋅ *g* for 19 h at 4 °C. The resulting pellet, containing purified OMVs, was resuspended in Hyclone™ sterile cell culture water (GE LifeSciences) and quantified using a Bradford protein assay as we previously described [35].

### Bacterial Susceptibility Assays

The antimicrobial activity of *B. thailandensis* OMVs was confirmed with a Kirby Bauer-like method. Briefly, a fresh culture of *S. mutans* was streaked heavily onto an agar plate, and 10 µg OMVs (adjusted to 10 µL with PBS) from each preparation were spotted directly onto the plate, using 10 µL PBS as negative control. The plate was incubated at 37 °C in an aerobic chamber containing 5 % CO_2_ under static conditions for up to 48 h at which time the plates were examined for growth inhibition.

### Growth Inhibition Assay

The ability of OMVs, HMNQ, or rhamnolipid to inhibit the growth of *S. mutans* was examined using a growth inhibition assay. HMNQ and rhamnolipid were purified from *B. thailandensis* OMVs at greater than 90 % purity as we previously described [35]. For testing OMVs, *S. mutans* were cultured overnight in 5 mL BHI broth at 37 °C. The suspension was further diluted in BHI broth and added to a 96-well plate (Costar) at 80 µL/well followed by treatments with 20 µL of PBS, native OMVs, or heat-inactivated OMVs at the same concentration. Heat inactivation of OMVs was achieved by incubation on a heat block at 80 °C for 2 h. For HMNQ and rhamnolipid, the purified compounds were dissolved in MeOH and added to a 96-well plate to a final concentration of 100 µM HMNQ, or 64 µg/mL rhamnolipid (based on previous dose determinations) and allowed to completely evaporate as previously described [35]. The overnight culture of *S. mutans* was diluted 1:1000 in BHI broth and added at 100 µL/well into a 96-well plate after evaporation. Optical density at 600 nm was monitored until the culture reached stationary phase.

### Planktonic and Biofilm Antimicrobial Assays

For planktonic cultures, *S. mutans* was grown overnight in 5 mL BHI broth without sucrose at 37 °C and 5 % CO_2_ under static conditions. One-milliliter cell suspensions were centrifuged at 3,500⋅ *g* for 10 min. *S. mutans* biofilm was induced using a modified protocol from previously published studies [55, 56]. Briefly, biofilms of *S. mutans* were grown on rectangular microscope coverslips in a semi-defined biofilm medium with glucose (18 mM) and sucrose (2 mM) (BMGS) for 3 days at 37 °C and 5 % CO_2_ under static conditions. The biofilm medium contained 58 mM K_2_HPO_4_, 15 mM KH_2_PO_4_, 10 mM (NH_4_)_2_SO_4_, 35 mM NaCl, 0.0001 % (wt/vol) FeCl_3_⋅6H_2_O and 0.2 % (wt/vol) casamino acids (pH 7.4), and was supplemented with vitamins (0.04 mM nicotinic acid, 0.1 mM pyridoxine HCl, 0.01 mM pantothenic acid, 1 µM riboflavin, 0.3 µM thiamin HCl, and 0.05 µM d-biotin), amino acids (4 mM l-glutamic acid, 1 mM l-arginine HCl, 1.3 mM l-cysteine HCl, and 0.1 mM l-tryptophan), and 2 mM MgSO_4_⋅7H_2_O [57–59]. The coverslips were vertically submerged in the culture medium, which was replaced daily. After 3 days, biofilms adhered to the glass surface were harvested by scraping off the coverslip and dispersing into 10 mL PBS. One-milliliter cell suspensions were centrifuged at 3,500⋅ *g* for 10 min. Cell pellets of planktonic and biofilm-associated bacterial cells were resuspended with 300 µL of 50 or 100 µg/mL OMVs, 800 µg/mL gentamicin, or PBS, respectively, followed by incubation at 37 °C and 5 % CO_2_ [60]. At 0, 3, 6, and 24 h, 70 µL of each cell suspension was mixed with 630 µL PBS and sonicated at 20 watts for 20 s. The sonicated cell suspensions were then serially diluted and plated for colony-forming units (CFU).

### Biofilm Assay and Confocal Microscopy

*S. mutans* was cultured overnight in BHI and diluted 1:10 in BMGS. Biofilms were cultured statically in uncoated 8-well chamberslides at 37 °C, with medium changed daily. To examine the antibiofilm activity of native OMVs, three-day biofilms of *S. mutans* were treated with 10, 50, or 100 µg/mL OMVs, or PBS for another 24 h. To examine the antibiofilm activity of heat-inactivated OMVs, one-day biofilms of *S. mutans* were treated with PBS, 100 µg/mL heat-inactivated OMVs or native OMVs for another 24 h. To examine the antibiofilm activity of HMNQ and rhamnolipid, one-day biofilms of *S. mutans* were treated with PBS, 200 µg/mL rhamnolipid, 300 µM HMNQ, or a combination of 200 µg/mL rhamnolipid with 300 µM HMNQ for another 1.5 h. HMNQ and rhamnolipid were purified from *B. thailandensis* OMVs with above 90 % purity as previously described [35]. Biofilms were then stained with LIVE/DEAD *Bac*Light fluorescent dye (SYTO 9/propidium iodide) (Invitrogen) and imaged on a Zeiss LSM 700 microscope with a 40x oil objective. Confocal *z*-stacks and simulated *xyz* three-dimensional images were generated using Zeiss’ Zen 10.0 software. Seven to ten image stacks were acquired from random positions within each well in order to cover a representative area of 200,000 µm^2^ of the total biofilm [41, 61]. For each *z*-stack, images were acquired at 1.0 μm intervals and analyzed using COMSTAT 2.0 [41, 62].

### Field Emission Scanning Electron Microscopy

*S. mutans* biofilms were grown on hydroxyapatite (HA) discs placed horizontally in 24-well microtiter plates as previously described [63–65]. Briefly, overnight bacterial cultures were grown in BHI and diluted 1:10 in BMGS. Two milliliters of the diluted culture were added to each well with HA disc. After incubating for three days at 37 °C in a 5 % CO_2_ aerobic atmosphere with medium changed daily, the HA discs were transferred into wells with PBS, 50 µg/mL, or 100 µg/mL OMVs for another 24 h. The HA discs were then washed with PBS and fixed in 2.5 % glutaraldehyde overnight at 4 °C. The fixed samples were dehydrated using increasing concentrations of ethanol, and desiccated with CO_2_ critical point drying. The samples were coated with carbon before imaging. Scanning electron microscopy (SEM) was performed with a Hitachi S-4800 high-resolution scanning electron microscope.

### Minimum Inhibitory Concentration Assays

Minimum inhibitory concentrations (MIC) of gentamicin cooperatively with or without OMVs for biofilm cells (MIC-B) and planktonic cells (MIC-P) were measured as previously described [60, 66]. For MIC-B, overnight *S. mutans* culture was adjusted to an OD_600_ of 0.5 and then diluted 1:1000 in BMGS. The bacterial suspension was added into a 96-well plate at 80 µL/well and incubated for 24 h at 37 °C and 5 % CO_2_. Pre-formed biofilms were then washed once with PBS and treated with a 2-fold serial dilution of gentamicin starting at 800 µg/mL, with or without 5, 10, 20 µg/mL OMVs for another 24 h. The treated biofilms were then washed with PBS to remove the treatments. To account for the detachment of viable cells within biofilms, fresh BHI was added into each well and incubated for 24 h. OD_600_ of the supernatant was measured before and after the 24-hour incubation. For MIC-P, overnight *S. mutans* culture was adjusted to an OD_600_ of 0.5 and then diluted 1:1000 in BHI. Eighty microliters of cell suspension were added to the wells of the 96-well plates. Twenty microliters of serial 2-fold dilutions of gentamicin (5x) were added in each well with the final concentrations ranging from 0.04 to 40 µg/mL. OD_600_ was measured before and after the 24-hour incubation at 37 °C and 5 % CO_2_. All experiments included three biological replicates and were independently repeated for three times.

### Statistical Analysis

Pairs of independent samples were compared with two-sample t-tests. Multiple comparisons were down with one-factor ANOVA. Pairs of curves were compared using a Chi-squared test [67]. This test enables calculation of single p-value for the null hypothesis that any two arbitrary curves were sampled from the same parent population. When multiple curves were compared, the Bonferroni correction for multiple comparisons was used to modify the cutoff for statistical significance [67].

## Data Availability

The datasets used and/or analyzed during the current study are available from the corresponding author upon reasonable request.

## Appendix

**Additional file 1:**

## Abbreviations

OMV: Outer membrane vesicle
EPS: Extracellular polymeric substance
LPS: Lipopolysaccharide
HMNQ: 4-hydroxy-3-methyl-2-(2-non-enyl)-quinoline
BMGS: Biofilm medium containing glucose and sucrose
CFU: Colony-forming unit
PI: Propidium iodide
HA: Hydroxyapatite
SEM: Scanning electron microscopy
eDNA: Extracellular DNA
MIC: Minimal inhibitory concentration
HAQs: 4-hydroxy-2-alkylquinolines
RGP: Rhamnose-glucose polysaccharide
LB: Lysogeny broth
BHI: Brain heart infusion broth
PES: Polyethersulfone
TCA: Tri-chloroacetic acid
