# Supplementary material for: Inhibition of Streptococcus mutans biofilms with bacterial-derived outer membrane vesicles
Source: BMC Microbiol. 2021 Aug 24;21:234. doi: 10.1186/s12866-021-02296-x (PMC8386047; doi:10.1186/s12866-021-02296-x)
Supplement: Supplementary file 1 — Additional file 1: [file 12866_2021_2296_MOESM1_ESM.docx]

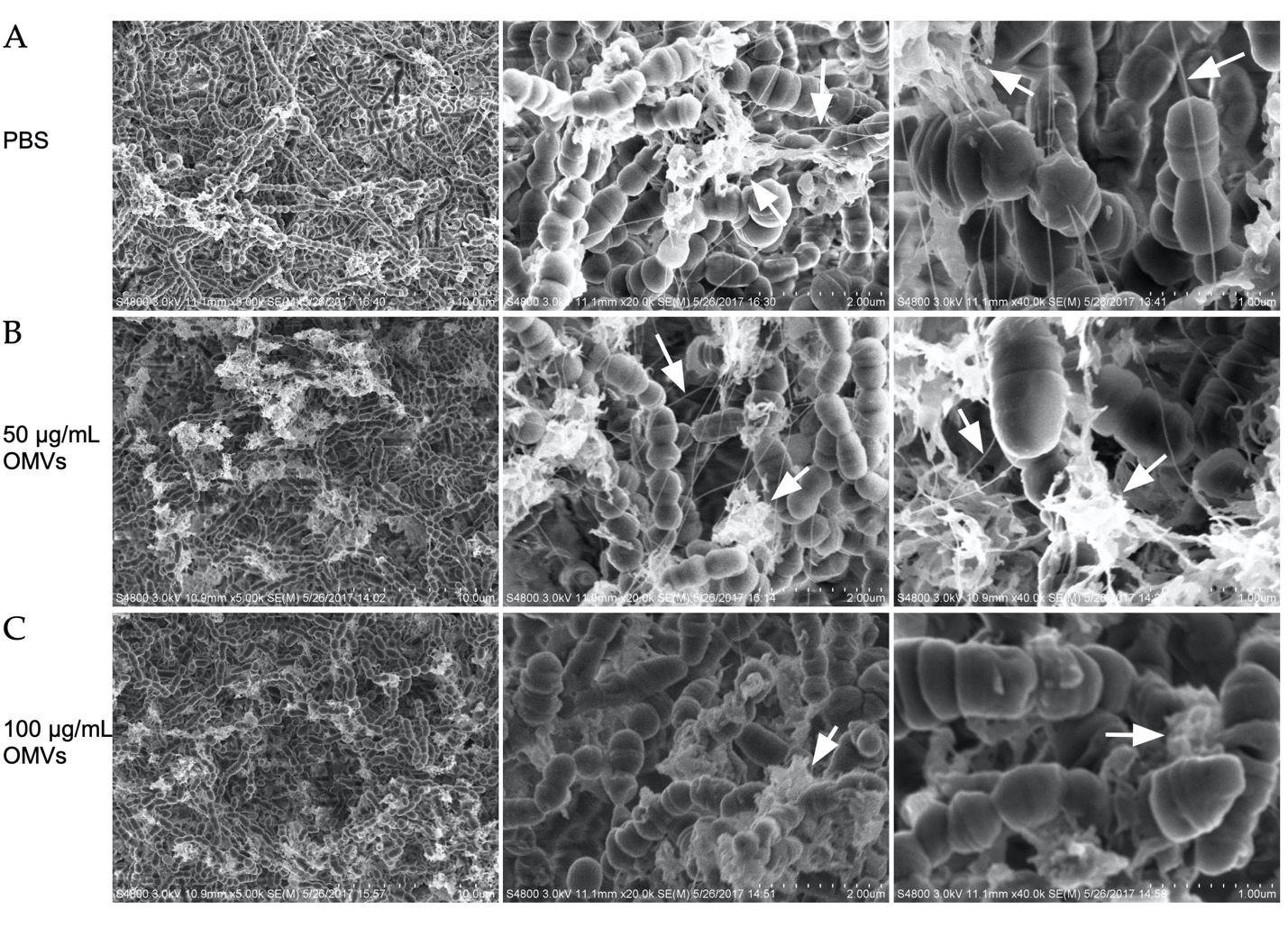


**Supplemental Figure 1.** OMVs alter biofilm structure and cellular morphology of *S. mutans*. *S. mutans* biofilms were grown on hydroxyapatite discs in BMGS medium for three days before treatment with **(A)** PBS, **(B)** 50 µg/mL, or **(C)** 100 µg/mL OMVs for another 24 hours, then imaged with SEM. Images were acquired at 5,000× (left), 20,000× (middle), and 40,000× (right) magnification with scale bars showing 10, 2 and 1 micron(s), respectively. White arrows point to extracellular material and structures.
